# Observations on Typhus Icterodes

**Published:** 1831

**Authors:** Robert J. Dodd

**Affiliations:** The U. S. Navy


### Art. IV. *Observations on Typhus Icterodes.*—By Robert J. Dodd, M. D., *of the U. S. Navy.*

There is, perhaps, no disease of a febrile character, which has excited more interest, or which is better calculated to create a spirit of inquiry than yellow fever. Some physicians, who have spent most of their lives in a tropical climate, and who have had ample opportunity of investigation, declare, that such was the instability, and incessantly changing character of this disease, that no one symptom would be discovered which was truly diagnostic—

while men equally high in rank, and eminent in their profession, announce, that they have found no difficulty in recognizing it or its varieties.

There is immediately before the development, and preceding an attack of this disease, a peculiar indescribable expression of the countenance, which cannot be mistaken by those who have witnessed it, and is frequently accompanied by chilliness, nausea, and extreme debility. After some hours, arterial reaction is developed, attended by an acute pain in the head, a hot skin, a general tenderness over the whole body, more especially about the epigastrium, with tension and fulness of the chest, and a dull heavy pain in the loins and hips. The eye is either engorged and glaring, or sad and watery; the tongue dried and white, with red edges; sometimes it is natural, or has the appearance of raw beef, being much elongated and flattened. There is also observed a peculiar pale-bluish, yellow or yellowish-white appearance of the skin around the ear, and on the upper part of the forehead. As the disease advances and febrile excitement is fully formed, the stomach ejects every thing that is taken, and not unfrequently a considerable quantity of bile is discharged in consequence of the continual pressure of the abdominal muscles on the liver and gall bladder. The skin is extremely hot and rough, giving to the hand when applied, a sensation so peculiar, that it is withdrawn immediately. The pulse is either full and hard, or small and tense, but not frequent. The pain in the head increases, and oftentimes delirium supervenes. The thirst is great, and respiration extremely laborious—whilst great uneasiness and anxiety prevail. Partial deafness ensues and the countenance exhibits the indescribable expression of despair, fixed and determined. These symptoms continue thirty-six, forty-eight, and often more than seventy-two hours, before a sensible abatement is distinguishable. A short time only intervenes when this remission is succeeded by symptoms of the most aggravated and appalling character. The stomach is violently contracted, and a dark fluid (in which hard flakes of the same substance are visible, mixed with ropy mucus and bile) is thrown up. Sometimes, however, no such fluid is ejected, either alone or mixed with other matter. The thirst is vehement and respiration difficult. The skin at this stage, acquires a bluish-yellow appearance; the deafness is almost complete; the pulse becomes weak and frequent; an exudation of a dark fluid takes place, from the mouth, nose, eyes, and ears, (which differs in no essential particular, from that substance which is discharged from the stomach, and which is also found in the cava and heart after death;) hiccough and convulsions follow, and death closes the scene.

Few cases occur in which the diversity of the symptoms is not greater than what has been already described, but it is impossible to define any certain set of them as characteristic of this malady, or to state in what precise time the different changes may take place—they would only mislead and confuse. In the majority of severe cases, no remission is observable, the disease commencing and terminating in a single paroxysm. In such cases, there was observed, loss of muscular action, with a rapid growth of the hair and nails, immediately before and after death. Now this I deem to depend on the electro-animal fluid gradually escaping from that power which sustains life, and which is about to separate from physical matter. The sudden development of this fact is, I conceive, owing to its being less under the control of vitality, being eliminated by the brain and ganglia, as so many distinct electro-galvanic batteries, and going off from the body by the nerves.

*Post mortem appearances.* The different organs presented uniform appearances, under similar circumstances, and differing only from accidental causes. The stomach and bowels presented no traces, or very slight marks of inflammation in the congestive character of this disease; the liver was found to be of a dark color, but not in a congested state; the lungs and spleen were also of a dark aspect; the brain was apparently much altered in its structure in those cases in which great pain and delirium had existed; but no signs of inflammation or congestion were visible. The cellular texture throughout the body was of a pale bluish yellow color, similar to that acquired by the skin. Is it not probable that the various elements of the different secretions unable to pass off, in consequence of the general derangement, have been cast, by an effort of the system, into the cellular texture? The mucous and serous membranes were quite dry. The muscular fibres possessed little tenacity.

The stomach, in the inflammatory variety, sometimes exhibited signs of extensive inflammation. In a few instances, the coats of that organ were partially destroyed. The intestines also shewed in a few cases, marks of inflammation. The liver and spleen were also distended with dark blood, but no alteration of structure was observed. The brain presented nothing unusual. The mucous membranes were very dry, while the serous were covered with a thick fluid. The cellular texture, throughout, presented the same appearance as in the other variety; as also, did the blood in the different cavities.

Besides the symptoms already enumerated, which characterize this distinction, the following are the diagnostic peculiarities of the two species. It is only by making these distinctions that we can arrive at a true knowledge of diseases; which is the more necessary, as their treatment is essentially different.

| *Inflammatory.* | *Congestive.* |
|---|---|
| 1. A peculiar suffused expression of the countenance, with a yellowish white appearance around the ear and along the upper part of the forehead. | 1. A peculiar contracted expression of the countenance, with a pale bluish yellow appearance around the ear and along the upper part of the forehead. |
| 2. The eye glaring and engorged. | 2. The eye sad and watery. |
| 3. The tongue furred, with a dark streak in the centre as natural. | 3. The tongue white and dry, or like raw beef, elongated and flattened. |
| 4. A pain over the eyes and deep in the head. | 4. A pain in the parietal region of the head, occasionally shooting through the eyes. |

| | |
|---|---|
| 5. The skin hot and rough, or dry and contracted. | 5. The skin hot and smooth. |
| 6. Great tenderness in the epigastric region. | 6. Tenderness over the whole body. |
| 7. Incessant vomiting. | 7. Occasional vomiting. |
| 8. Bowels obstinately constipated. | 8. Bowels sometimes loose. |
| 9. Pulse full and hard. | 9. Pulse small and tense. |
| 10. Respiration laborious. | 10. Respiration moderate. |
| 11. The countenance wearing an indescribable expression of despair, fixed and determined. | 11. The countenance wearing an indescribable expression of conscious hopelesness. |
| 12. A dark fluid is ejected from the stomach, with seeming pain and distress. | 12. A dark fluid is thrown up with an expression of relief. |
| 13. Delirium, with partial deafness. | 13. Consciousness, with partial deafness. |
| 14. Coma, hiccough, and convulsions. | 14. Sensible, with a sinking of the vital powers. |
| 15. Death. | 15. Death. |

In relation to the causes of yellow-fever, much difference of opinion exists among practitioners. Whilst some consider it a disease possessed of a peculiar poison, capable of being carried to different and distant parts, others, and of late the majority of the profession, have discarded the opinion of yellow-fever being a contagious disease, and suppose it to have a domestic origin. Many have attributed to marsh-miasmata the power of generating this disease; but as no one has proved their existence, and other causes can be assigned, I make no hesitation in asserting, that marsh-miasmata, possessing specific virtues in creating fevers, do not exist but in the imagination.

Excessive fatigue, night-watching, fear, mental depression, the immoderate use of ardent spirits, eating unripe fruit, or low diet with a deficiency of salted or seasoned provisions and vegetables, predispose to, and exposure to a high range of temperature, give rise to this affection.

It is evident that few cases occur among those persons who have not been exposed to the influence of a burning sun. It appears essentially necessary to the full formation of this fatal malady. The system is more easily deranged in the forenoon, than at any subsequent part of the day; at that time a greater portion of the body is acted upon by it, whereas, at noon, the sun is nearly vertical, and a large hat or umbrella, defends the person from its scorching rays; and by the time it would again throw a lengthened shadow, the body becomes less susceptible to its action. I know of no instance where the disease was communicated from one to another, nor do I believe it to be, in any way, contagious. Like all other diseases of a violent character, the excrementitious matter is very offensive, and is sufficient to excite disease, if not removed, and proper attention paid to cleanliness.

In the treatment of this malady, great attention should be paid to the state of the system. When the pulse is full and hard, the eye engorged (the vessels of the conjunctiva injected with red blood) and glaring, the pain in the head severe and deep seated, the tongue furred, the skin hot and rough, and respiration laborious, bleeding is essentially necessary, and should be resorted to, boldly, even in the advanced stage of the disease. I have taken, on the third day, when I had not seen the patient before, as much as fifty ounces of blood, before any sensible benefit was derived from the operation, and then only a slight mitigation of the pain and softness of the pulse took place, which, in a short time, was followed by more evident changes. In the abstraction of blood from the arm, two things are to be strictly observed: that the orifice be made large, to enable the blood to escape in a bold and rapid stream: that the blood be allowed to flow until an impression is made upon the system, and the blood changes to a lighter color as it escapes from the orifice. If blood is drawn, in any quantity, after this change, the advantages which might otherwise acrue to the system, are entirely lost, and the patient very much debilitated. When a less quantity is abstracted, the beneficial effects are not so great. One large bleeding is all-sufficient,

Sometimes cases occur where reaction takes place so slowly, and the depression is so great, that frequent small bleedings, from half an ounce to two ounces every hour or two, until perfect reaction had been fully developed, have been followed by the happiest results. If, however, the vessels of the conjunctiva are filled with dark blood, the eye sad and watery, the tongue white and dry, or like raw beef, the pulse at the same time small and tense, blood should not be drawn either at the commencement or any subsequent stage of the disease; nothing would tempt me to do so, if the eye and its vessels were in that condition, even though the pulse were full and hard. The blood which I have seen drawn under these circumstances, by those who have advocated blood-letting in the first twenty-four hours of the disease, without regard to the condition and state of the system, would not coagulate, possessed very little fibrin, and was deficient in saline matter. In the inflammatory variety, the blood drawn, contained less fibrin and saline matter, than when drawn under different circumstances, but more than was found in the congestive form of the disease. If great pain in the parietal region of the head existed, the natives of the middle district of Cuba, denominated it *taberdalia*, and considered the patient in a hopeless condition.

The dermoid capillaries cease to perform their usual functions, and the skin becomes extremely hot on the accession of the febrile paroxysm. When the cuticular surface was rough, or dry and contracted, I employed lime-juice, with which I sponged the whole body with the greatest possible benefit; indeed it appeared to act with specific advantage upon the capillaries, in lessening the heat, smoothing the skin, removing stricture and restoring their action. Vinegar and water also proved of considerable advantage, in the same manner, as also water applied in the form of affusion. Wetting the sheets with vinegar and water, repeating it as often as necessary, was attended by the most grateful sensations and beneficial results. I have seen cases of the most alarming nature, yield to this course of treatment.

The stomach being the great central organ by which the body is supplied, it necessarily follows, that whatever has a tendency to weaken its operations, or derange its functions, predisposes to disease. A disordered condition of its functions cannot exist any length of time, without involving other organs to a greater or less degree, though its action may be so insidious as to be scarcely noticed. Nor can any part, however remote, be affected, to any extent, without a sensible impression upon it. It is through the medium of its great and important sympathies, that we are enabled to combat the diseases incident to the human body; and when we are deprived of that medium, our efforts are truly limited. It exhibits derangement from the commencement, which increases as the disease advances. It has been supposed, by many, to be the seat of the disease, and to be uniformly in a state of inflammation. But although it may be inflamed, occasionally, as any other organ; although it may show signs of extensive disorganization, on examination after death; although all appearances may warrant the conclusion, that inflammation has existed in this as in other organs; still it does not prove, that this or that organ is the primary seat of the disease, or that inflammation in either of them is necessary to the formation of it. In numerous instances, no local signs of disease have been traced—nothing that is calculated to lead the most sanguine to conclude it to be of a symptomatic character. In irritation of the stomach, I have resorted to emetics so late as the third day, which have never failed to allay its spasmodic action almost immediately. It appears that the irritation of the stomach depends upon the derangement of its nervous action; and whatever will produce a strong impression upon that organ, is sufficient to allay the morbid excitement. The articles of that class which I used, were the ipecacuanha, sulphate of copper, and the sulphate of zinc; fifteen grains of ipecacuanha, with fifteen or twenty grains of the sulphate of zinc, or from six to ten of the sulphate of copper, was the general dose administered. After the operation of the emetic, a little arrow-root was given, which the stomach invariably retained.

Diaphoretics were likewise advantageous in the febrile stage, and their operation was much assisted by the previous action of emetics, and appropriate applications to the skin. Most of the cooling diaphoretics produced the desired effect, but I found none so decidedly salutary in this malady, as the pulvis antimonialis, given in doses of eight or ten grains, every hour. Diluent drinks were likewise very serviceable, acidulated with lime-juice.

The bowels, were invariably, in a constipated state in the inflammatory, and mostly in the congestive forms of this affection. In both kinds, relief was afforded by the same mode of treatment. Injections of sea-water, or salt and water, did more good than all the purgatives together; they were continued till the bowels were completely evacuated. There was, oftentimes, an atony of the bowels, which was so great, that not unfrequently, several quarts were injected into the intestinal canal, before any effect was produced; and then, apparently, only by the bulk and gravity of the fluid, without any action of the bowels themselves to pour it out. I have, in some cases, despaired of ever being able to accomplish any thing by this means. By perseverance, however, the dark, hard, and offensive matter which they contained, came away, and they gradually recovered their functions, to my great gratification. I have every reason to suppose, that some portion of the sea-water is absorbed. It allays the sensation of thirst effectually. In most cases the continuance of the injections have been followed with good effects. After the bowels have been freely opened, and the irritation of the stomach has subsided, purgatives may be given. Twenty grains of calomel, with ten or fifteen of jalap, or with a small quantity of some neutral salt, answered not only to affect the bowels, but likewise the liver. If the atony had been great, I gave the drastic purgatives, singly or combined with others. The following formula was one I sometimes used in such cases.

R. Aloes socot. 1-2 dr.
G. Gambogii grs. 10.
Tart. Ant. grs. iij.
Sub. Mur. Hyd. grs. xv.
Ol. Croton gtts. v.
P. G. Arabic et
Syr. simp. a a q. s.
Misce et fiat in pilulas, xx.

Of which a dose was four, or as many as circumstances required. Small doses of the neutral salts were given during the whole course of the disease, to keep the bowels in a free state.

The liver being more or less intimately associated in function with the stomach and skin, is much deranged in this malady; sometimes it is in a conjested state. There are no remedies which act alone upon the liver, but there are those that produce impressions primarily on other organs, and those which affect generally the glandular system. It is not essentially necessary that this organ be wholly relieved before a crisis takes place; yet it is important, that total relief be afforded as soon as possible, that the treatment subsequently may be simple, and restoration to health speedy. The muriatic, and nitro-muriatic acids were useful in removing obstructions. The application of cups and blisters to the sides proved beneficial.

The brain and nerves constitute the grand chain of connection between all parts of the system, and besides performing an independent function, they are intimately blended with the functions of every other part. When one of them is in a state of lesion, the brain is secondarily affected, and will tend to keep up the disease first induced, leading us often times to suppose that it was primarily impressed. In the congestive form of this disease, when the pain in the head was severe, the application of cold water, or vinegar and water, and in the inflammatory variety, that of cups, leeches, and blisters, gave relief to the scalp. From the application of blisters, I have never known any advantage; they have only created pain and uneasiness.

When the pain began to abate, and the pulse began to soften, the sulphate of quinine or sulphate of cornine was administered in large doses, usually in conjunction with powdered cinnamon or ginger. If the remission did not take place in less than forty-eight or seventy-two hours, and had not been brought about by previous treatment, from twenty to thirty grains were given every twenty minutes or half an hour, until a hundred or a hundred and fifty grains had been taken, then a less quantity at longer intervals. On the contrary, if the remission took place in twelve or twenty-four hours, and had been produced by active treatment, from fifteen to twenty grains administered every hour, until eighty or a hundred grains had been taken, was all that was required. Bark in substance, or some other tonic was employed as soon as the fever was broken, and continued till health was perfectly restored. The cornine, in small doses, was preferable, in some cases to the bark. When either of these remedies was given in large doses, the other, or some astringent tonic followed in small doses, because a small dose of a medicine which had been administered in large doses, was not efficacious, whereas, another produced all that was expected. In some cases of the congestive form, the mineral tonics, particularly the iron, were more beneficial than the bark. The tonic remedies were administered after the first exacerbation, and in no instance as yet have I witnessed their failure.

In connection with the medicinal treatment, attention was also paid to articles of diet. I have always found it better to supply the stomach regularly with a quantity of food of a proper kind, sufficient to keep it in action, thereby assisting the operation of medicine, and preventing unpleasant symptoms. Arrow-root, tapioca, sago, &c., were very grateful to the stomach, and of these, the arrow-root was decidedly preferable. Barley-water, flax-seed tea, rice water, &c., were used as diluent drinks. Such articles as contained a considerable quantity of nourishment, and were palatable, were preferred during convalescence, given in moderate quantities, at stated periods. After the patients recovered a little strength, much good was derived from sea-bathing and the exercise of riding, taking care not to be exposed to the sun, night dews, or morning fogs. The bowels were kept open by small doses of the neutral salts. The patients lived principally on the meat of young animals and vegetables, avoiding the use of beef as much as possible. It is a singular fact, that all parts of the bullock which lie within an inch from the surface, are unfit for use, being hard and tough.

A few cases are subjoined, the history of which was taken at the time, with an account of the mode of treatment.

J. L., a cooper from the United States, had been in the West-Indies about twenty days, had been cautioned not to expose himself either to the sun or night dews, but paid no attention to the friendly advice given him, until he perceived his strength was not sufficient for him to continue his ordinary avocation. Late in the evening, 24th June, complained of being cold and chilly—told his landlady his head ached, and desired medicine—took some oil and retired to bed—began to vomit—became feverish—looked wild, and talked very incoherently—was very restless during the night, and breathed unusually hard. In the morning, 24th—continued vomiting—was delirious—spoke of suffering from pain in the head and stomach—had much fever, and his skin was hot and rough—was extremely thirsty—eyes engorged with red blood and protruding—tongue very much furred and thickened, of a brown color, and had a dark streak in the centre—bowels had not been open for more than three days—pulse full and hard, resembling the turning of a large cord under the fingers—breathing performed with signs of oppression—face suffused, and wore a peculiar expression, with an unusual appearance of the skin on the forehead and around the ear—had passed but a small quantity of urine, which was of a high color, and deposited a large quantity of red matter on standing a few minutes—pain in the loins and thighs excruciating.

A vein in his arm was immediately opened, and sixty ounces of blood drawn before any change was observed in the pulse, or in the blood, when it was thought advisable to bandage the arm—was then washed with lime juice, and as the vomiting continued, an emetic was administered, consisting of fifteen grains of ipecacuanha and twenty of the sulphate of copper, which operated almost instantaneously—bowels were then evacuated by means of cold sea-water injections, and which were continued till the larger intestines were supposed to contain very little fecal matter—pulse became softer—pain in the head less severe—respiration more free—skin not so hot or rough—eyes more moveable—the vessels less engorged—irritation of the stomach wholly removed, and the patient generally much improved—cups applied to the temples and sinapisms laid on the calves of the legs and soles of the feet—a dose of calomel and jalap administered—sheets wet with vinegar, and water and to be repeated when they became dry—gave a little thin arrow root—eight grains of the pulvis antimonialis to be taken every hour—was allowed to drink rice-water—towards evening, the symptoms gradually abated, the injections having been administered every two hours—signs of remission shewed themselves on the 25th, at night—twenty grains of quinine, mixed with some powdered cinnamon were given every half hour, for three hours, and ten grains every hour for six hours—then altogether omitted. Proper nourishment, small doses of cornine, and the bark in substance completed the cure.

J. F., a girl fourteen years of age, native of the West Indies, had been on the beach searching for shells, in the middle of the day, 24th June, exposed to the hot sun. On her return home, she complained of a headach, and went to bed, in the evening, had considerable fever, pulse small and tense—head very painful—eyes sad and watery—face pale, and countenance exhibiting an expression of great anxiety and distress—spoke wildly, and was somewhat delirious—tongue white and furred—vomited a great quantity of watery fluid, tinged with bile—much debility existed—sharp pain in her head, over her ear, sometimes in the eye of the same side, and in the back—started when pressure was made on the abdomen—skin hot and sore to the touch—bowels had been loose the preceding day—respired freely, but felt somewhat oppressed—gave her an emetic of Ipecacuanha and sulphate of copper, twelve grains of the former with eight of the latter—salt-water injections administered, and directed to be continued every two hours—took a dose of calomel and jalap—sinapisms applied to the wrists and ankles—towels wrung out of cold water, placed about the head, to be removed as often as necessary—during the night, drank water, in which tartarized antimony was dissolved. In the morning, 25th, was somewhat relieved—remedies continued. In the evening, a remission took place—six grains of quinine were ordered to be taken every hour, until morning—26th—was entirely free from fever—quinine was omitted, and small doses of the precipitated carbonate of iron substituted in its place—with a free diet and gentle exercise, recovered in a few days.

Mr. P. had been in a tropical climate several months. 2d July, was on horseback several hours, exposed to the sun, in the morning, without eating breakfast—was seized with pain in the head and chilliness—complained of weakness of the stomach and vomiting, which were succeeded by flashings over the face—much pain in the back and loins, soreness of the abdomen, pain in the side—soon after, fever took place—eyes became engorged with red blood and glaring—tongue dry and red along the edges—had a peculiar expression of the countenance when spoken to—suffered from a pain shooting into the eyes—pulse quick and full—skin dry and hot—bowels constipated—respiration laborious, with great oppression. Thirty-five ounces of blood were taken, when the pulse became softer—respiration more free—bowels were opened by injections of salt-water. An emetic, consisting of fifteen grains of ipecacuanha and four of sulphate of zinc was given, and afforded immediate relief from those symptoms arising from a disordered stomach. Affusions of cold water and diluent drinks brought about an abatement of the symptoms. The sulphate of quinine was directed to be used in doses of ten grains every hour, for eight hours. The subsequent use of Colombo, gentian, and quassia, with proper diet restored him to health.

A. S., a young lady, native of the United States, had resided in the Island of Cuba more than two years. 2d of July, in the evening, was seized with fever. On the morning of the 3d, complained of a severe pain in the head and soreness of the whole body; eyes were sad and watery, and unable to bear the light; tongue white and dry, skin hot and smooth; countenance bore a peculiar expression; the skin along the upper part of the forehead and around the ear, appeared of a paler color; bowels constipated. There was slight nausea, but no vomiting; gave her a dose of calomel and jalap, and directed injections of warm water, to which were added oil and table-salt. Sinapisms were applied to the feet; the pulvis antimonialis, in doses of six grains, was given every hour, with diluent drinks; occasionally a spoonful of arrow-root was taken. On the morning of the 4th, a remission took place. The quinine was administered in doses of ten grains, with cinnamon, every hour, for three hours, then six grains for several hours. A decoction of serpintaria was ordered, and food of the most nourishing kind, taken, prepared in a palatable manner. Gentle exercise in a volante, and bathing, were recommended. In a short time she regained her strength and spirits.

J. G., a fisherman, native of Cuba, was in the habit of assisting in work on board of vessels, whenever an opportunity occurred, during which time he received a daily allowance of spirits. Having been at work several days, was attacked, on the 25th of September, with pain in the head and fever, while exposed to the influence of the sun, on the ship's deck; was carried on shore, and immediately made application for relief; pulse full and strong; eyes glaring, and the vessels engorged with red blood; tongue dry, white, and furred, with yellow edges and a brown streak in the centre, sharp at the point and broad at the base; has a peculiar indescribable expression of the countenance, with a whitish-yellow appearance of the skin on the forehead and around the ear; complained of a tightness of the chest and a difficulty in breathing, soreness of the abdomen, and a deep, heavy pain, in the back and head; bowels were constipated; had not passed urine, in any quantity, since yesterday; skin dry and hot; pain through the chest, neck, and head, almost insupportable. Blood was immediately drawn from the arm; the body sponged with vinegar; bowels opened by the use of sea-water injections; cold water was dashed on the patient several times, with decided benefit. Ten grains of the pulvis antimonialis were given every hour, and the injections repeated; drank nothing but cold water, acidulated with a little vinegar. Bruized onions and garlic were applied to the feet, and a blister to the nape of the neck. In fourteen hours, an abatement took place; fifteen grains of the sulphate of cornine, with as much powdered ginger, were given every hour, for six hours; then, by the use of small doses of the sulphate of quinine and the serpentaria, the cure was effected.

W. S., a carpenter and cooper, had been in the West Indies upwards of six weeks; drank freely of spirituous liquors, and worked hard; was very fond of fruit, and eat little else; was taken down with fever, 28th of September, and sent for a physician. On the 1st of October, in the evening, I saw him, in consultation with the medical attendant, a native of that place, who made no hesitation in declaring that he could not live twelve hours; was speechless; tongue swelled and thrust out of the mouth; ejected a dark fluid from the stomach; vessels of the eyes engorged with dark blood; pulse full and slow; cathartic medicines that were taken, had not operated; hands were every now and then pressed against the side of the head; the nurse stated he spoke about the pain in his head being very great the day previous; skin was exceedingly hot, rough, and so contracted, as to resemble dried leather; bowels had not been open from the commencement of the disease; countenance had an expression truly distressing, expressive of a state of sullen despair; skin of a pale bluish-yellow on the forehead. An emetic was given, and his mouth washed with pine-apple juice. The body was bathed with lime-juice, and the sheets wet with vinegar and water. Cups were put on the temples, and bruised onions, with mustard, applied to the feet. The following cathartic was then administered:

R. Aloes grs. vi.

G. Gambog gr. i.

Calomel grs. x.

Ol. Croton gtts. ij.

P. G. Arabic, q. s.

m. et ft. in pilulas.

When the bowels were opened, the pulvis antimonialis, in small doses, every half hour, with diluent drinks, was given, as soon as circumstances permitted. In the morning of the 2d July, had improved wonderfully; could understand what was said to him, and reply, by motions with his head; skin was quite yellow, but not so rough as yesterday; medicines and injections continued. At 12 o'clock, the symptoms had so far abated, as to admit the use of the quinine, of which twenty grains were administered every twenty minutes, for two hours. Afterwards, the bark, every hour, with a generous diet and moderate exercise, soon enabled the patient to attend to his ordinary occupation.

J. N., a cigar manufacturer, native of the West Indies; could receive no information concerning the causes, or in what way the disease made its appearance. On the first of October, found him laboring under a violent fever; pulse full, but not hard; eyes watery, the cornea flattened and irregular; face much suffused, with an appearance of the skin wholly different from any I had before witnessed; it appeared yellow, spread over with bluish white spots, about the size of a shilling; horror and dismay were depicted in the countenance; tongue much swollen, and hung out of the mouth, covered with a black crust a quarter of an inch thick, and quite hard; vomiting continually a dark substance, which resembled clotted blood mixed with bile; was insensible to all that passed, and several times applied his hands to his head, indicative of suffering. The expressed juice of a ripe pine-apple, was given in tea-spoonfuls, which allayed the vomiting in a few minutes. Sea-water injections were used without success. An injection was then administered, consisting of two ounces of castor oil, and four of the spirits of turpentine, mixed with a quart of warm water, which produced a copious tar-like evacuation. The salt water was then continued, with evident benefit at stated periods. The skin was washed with lime-juice, and afterwards with vinegar. Cups were applied to the temples, and blisters to the neck. Little alteration took place for several hours. A strong infusion of capsicum and vinegar was rubbed on the extremities. A dose of croton oil produced a number of stools; became sensible of relief, and endeavored to speak. It was thought advisable to wash his mouth with lime-juice, and remove the crust from the tongue; took warm diluent drinks with eagerness, and gradually acquired the power of speech; signs of remission showed themselves, and the quinine administered in doses of twenty-five grains every half-hour, for four hours; subsequently, the bark and iron were alternately administered, until medicine was no longer required. Proper diet, sea-bathing, and gentle exercise, restored him to health in a few days.

S. R., a laundress, forty-five years of age. They stated that she had been washing, on the 22d of October, at the river, in the sun, and had been wet by a shower of rain, but did not change her clothes till evening; that she then felt unusually weak and feeble, and experienced a sensation of heaviness in the head; that she went to bed, and rested badly during the night, and the following morning was attacked with fever; that she had taken several doses of medicine, without being affected by them. On the 25th, fever continued violent; pulse full, hard, and strong; vessels of the eyes engorged with red blood, the cornea appearing unusually convex; skin on the forehead of a pale yellowish color; tongue thick and furred, with a black crust upon it; voice inarticulate; teeth encrusted; respiration painful and laborious; was vomiting, incessantly, a dark fluid, resembling coffee-grounds; skin hot, rough, and unpleasant to touch; bowels had remained obstinately constipated, for the last four or five days; pain in the head excruciating, as also was the pain in the loins and legs distressing; abdomen could not be pressed upon, without producing unpleasant symptoms; urine had been passed in small quantities, and of a high color; had not slept since the commencement of the disease; cannot comprehend what is said, unless spoken to in a very loud tone, and is constantly endeavoring to get up.

Blood was drawn to the amount of forty or fifty ounces, an emetic given, and the body bathed with fresh lime-juice. Blisters were applied to the nape of the neck and breast, and sinapisms to the feet; injections of sea-water were administered repeatedly, during the day, which gave great relief. The pulvis antimonialis ordered every hour, and rice-water to be drank freely. In the evening, the violent symptoms began to abate; the blisters were dressed, and sinapisms omitted. Thirty grains of quinine were then given every half hour, for three hours, and the bark, with wine, continued, until the patient was out of danger.

W., a physician from the United States; had been in the West-Indies only a few days; eat a considerable quantity of fruit; paid no attention to the admonitions of his friends. 23d of October, was attacked with fever; pulse small and soft; eyes engorged with dark blood, and glaring; felt a severe pain in the side of his head; bowels had not been open for two or three days; countenance pale and contracted; tongue dry and furred; skin dry, and somewhat rough; respired with considerable difficulty, and complained of great soreness about the chest and arms. Gave him a dose of calomel and aloes, which produced several copious evacuations; was directed to drink freely of rice-water, and take eight grains of the pulvis antimonialis every hour. In the evening of the 24th, a remission took place, and the sulphate of quinine, in doses of fifteen grains every hour, for four hours, and four grains for six hours, were all that was required, with moderate diet and gentle exercise, to complete the cure. Small doses of neutral salts were taken, as occasion required.

There is nothing so well calculated to prevent sickness on board of vessels, as cleanliness. The practice of washing decks every morning, when the weather will admit of it, is attended with personal benefit, as well as calculated to cleanse and preserve the vessel and what appertains to her. Each individual of the watch, pulls off his shoes and stockings, (or should do so,) and rolls up his trowers, before he commences. The custom of washing the feet, is not of modern adoption; and that of washing the face and hands, is practically acknowledged, by all, to be of great utility. I recommended bathing, in port, at least once in twenty-four hours; when at sea, to strip and dash water on each other, in the morning, before washing decks, or in the evening. The men became extremely fond of indulging in so salutary a practice. A number, who wore their shoes and stockings when wet, from indolence or necessity, were afflicted with diarrheas, rheumatism, catarrhs, &c. The wearing of flannel contributes greatly to the health of the crew, as also did the domestic cotton, manufactured with a nap on one side, which was worn next to the skin, which I believe is preferable to flannel. No clothes that were wet, should ever be allowed to be put away in that state. In the evening, they wore jackets of cloth, and were directed to keep them on during the night. Changing clothes twice a week, and as often as they became wet, tended to prevent unpleasant consequences. Much depends upon the disposition of commanders, in consulting with surgeons, on the means to be used in preserving the health of the men. Unless such a disposition is shown, the efforts of surgeons are defeated, large sick-lists present themselves, and the crews are generally feeble and depressed. Good water greatly assists in the preservative of health, and much care should be taken to attain it. That which is procured from rivers, will keep longer and better than that obtained from springs, and seldom contains so large a quantity of vegetable matter. The casks should be well cleansed, and the bungs allowed to remain out some days. In the West Indies, when the weather is extremely sultry, the consumption of water is necessarily greater, and the very mention of an allowance, causes such an excitement, that twice the quantity of water is drank, and thirst unallayed.

I have observed, when the water has been placed in the scuttle-butt, regularly, without naming an allowance, and refilled as often as required, the consumption has not been more than one gallon per man, a day, in the warmest weather, and seldom more than three quarts; a less quantity will hardly suffice. Music is well calculated to cheer the mind and enliven the spirits. It is really gratifying, to see the sturdy mariner, who has toiled all day, shake off his weariness and strike nimbly his foot to the sound of the violin.

Supplies of fresh provisions, while in port, add much to the preservation of health, but every other day is sufficiently often, while on the intermediate days the crew should receive their regular allowance of salted provisions. The bowels of sea-faring men, generally, have a tendency to be constipated, except after the use of fresh provisions. I have had as many as twenty, on the list of fevers and diarrhea, in two days, when they have been at work, exposed to the sun, on shore, and supplied with fresh provisions; but from proper treatment, were returned to duty in as short a time.

To prevent the formation of bilge water, and keep the ship clean, water was let in twice a day, in sufficient quantities to mix with that already in the vessel, which could not be removed, by the pumps, owing to the formation of that part of the vessel, and pumped off again. Many merchant vessels are possessed of no means to let water into them, and consequently the action of the water on the wood, creates an offensive odour, which in rough weather cannot be endured for any length of time without creating sickness. This would not be the case if the water was frequently changed. Some entertain an idea that no water should be admitted, as if it had a tendency to make a vessel unhealthy. Having had upwards of two years' experience in that particular, in a tropical climate, the cleanliness of the vessel, and healthiness of the crew, warrant me to say, that no bad effects attended the practice. In large vessels, the lower decks are more frequently rubbed with dry holly stones, with a view to cleanse them without the use of water; but it will occur to every impartial one, that such a practice is attended with injurious consequences, and defeats the very object for which it was intended. In the first place it is injurious, by constantly wearing away the decks, and the tops of mess chests; secondly, by reducing the sand to very small particles, the men at work necessarily inhale more or less of it, which produces unpleasant symptoms. A number of men were attacked with pain in the chest, cough, and expectoration of pus; the cause of which I would attribute to nothing but a change of weather and the inhalation of this dust. The washing of the lower decks in the morning, at least once a week, and drying them by means of stoves, hot shot, and heated sand, is attended with less injurious effects. Sprinkling of sand on the floor in the evening, will be sufficient to protect the deck during the night. Sprinkling with the chloride of lime, mixed with water, before washing the decks, and permitting it to dry, cleanses and whitens them. It also purifies the air, and renders the vessel wholsome. The chlorine being one of the electro-negative substances, combines like them, with most other substances, and in that manner renders the obnoxious vapours and gases nugatory; but its constant use and disengagement, in large quantities, is extremely injurious. It has been supposed capable of preventing yellow fever; but I am constrained to say, my experience and observations do not establish such a conclusion. Vessels, on board of which it has been regularly and constantly employed, were not more healthy, nor as much so, as those in which it was seldom used. If it was capable of preventing yellow fever, I should suppose it would likewise have prevented other fevers. On board of one of our public vessels, the surgeon officially informed me that upwards of forty cases of bilious fever had occurred, yet he persevered in its use, and deemed it sufficiently powerful to prevent yellow fever. That malady shewed itself on board of vessels where it was applied continually, and in one which appeared perfectly sweet and clean, which renders it more apparent, that it does not arise from any thing peculiar to the vessel. Hence, the evidence in favor of the chloride of lime being an article of value in rendering an impure atmosphere respirable and wholsome, but by no means sufficient to prevent yellow fever.

